# Serum beta2-microglobulin levels are highly associated with the risk of acute ischemic stroke

**DOI:** 10.1038/s41598-019-43370-9

**Published:** 2019-05-03

**Authors:** Sen Qun, Fuyong Hu, Guoping Wang, Juncang Wu, Qiqiang Tang, Ji Zhang, Zhengxu Chen, Xiaoqiang Wang, Qiuwan Liu, Wei Ge

**Affiliations:** 1 Department of Neurology, The First Affiliated Hospital of the University of Science and Technology of China (Anhui Provincial Hospital), Hefei City, Anhui China; 2 Department of Neurology, The Hefei Affiliated Hospital of Anhui Medical University, Hefei City, Anhui China; 3 School of Public Health, Bengbu Medical College, Bengbu City, Anhui China; 4 Department of Neurology, The Affiliated Hospital of Xuzhou Medical University, Xuzhou City, Jiangsu Province China

**Keywords:** Stroke, Neuroimmunology

## Abstract

Inflammation is considered an important mechanism of cell death or survival after ischemic stroke. As an important marker of inflammation, the role of β2-microglobulin (β2M) in acute ischemic stroke is unclear. We investigated the relationship between serum β2M and the risk of acute ischemic stroke (AIS). Patients with AIS (202 cases), intracerebral hemorrhage (ICH, 41 cases), and healthy controls (253 cases) were recruited. Clinical and biochemical characteristics were collected. We used three binary logistic regression models to evaluate the correlation of β2M with the risk of AIS. Furthermore, we investigated the relationship between serum β2M and the National Institute of Health Stroke Scale (NIHSS) score, the Trial of ORG 10172 in Acute Stroke Treatment (TOAST) subtypes, and the Essen Stroke Risk Score (ESRS) in patients with AIS. Our results showed that serum β2M levels in patients with AIS were much higher than those in patients with ICH and in the control subjects. Individuals with higher levels of β2M had higher odds of AIS. Moreover, serum β2M levels were significantly and positively correlated with ESRS. In addition, the levels of β2M were varied with different subgroups of AIS (TOAST classification). Serum β2M is highly associated with the risk of AIS.

## Introduction

Beta2-microglobulin (β2M) is a small molecular protein (11.8 kDa) that is secreted from all nucleated human cells with daily synthesis rates ranging from 2–4 mg/kg per day in healthy individuals. Serum β2M is metabolized almost exclusively (99%) from the kidneys and remains stable at 1–3 mg/mL^1^. As one of the classical low-molecular-weight markers of kidney function, the serum β2M level is highly inversely associated with the glomerular filtration rate. At the same time, serum β2M concentration is often influenced by many nonrenal determinants, such as systolic blood pressure, gender, total cholesterol, inflammation, and smoking^2^.

β2M is a critical component of the major histocompatibility class I (MHCI) complex heterodimer that presents intracellular antigens to cytotoxic T cells^3^. β2M dissociates from the cell surface or releases from inside of the cell and then sheds into the blood. The serum levels of β2M are associated with a variety of autoimmune diseases, tumors, infectious diseases, and renal disease^4^. However, recent studies have shown that β2M is also associated with a high risk of peripheral artery disease (PAD), cognitive dysfunction, and prevalent asymptomatic carotid atherosclerosis^5–7^. Therefore, more evidence indicates that serum β2M is not only a marker of kidney function but also has other functions in inflammatory diseases.

Recent studies have shown that inflammation is an important mechanism that determines the death or survival of cells after cerebral ischemia^8^. At the same time, the knockout of MHCI in animal studies of cerebral ischemia showed a neuroprotective effect^9^. As a critical component of MHCI, the role of β2M after acute ischemic stroke (AIS) remains unclear. A study showed that plasma β2M is associated with the occurrence of major adverse cardiovascular events (MACE) in patients with asymptomatic carotid atherosclerosis^5^; moreover, plasma β2M is an informative risk marker for both coronary heart disease (CHD) and stroke in postmenopausal women on hormone therapy^10,11^, and high levels of β2M were associated with an increased risk of ischemic stroke among women^12^. However, there are few clinical studies about the characteristics of β2M in patients with acute cerebral infarction, and a small amount of clinical data discussing the correlation between the levels of serum β2M and the risk of AIS/recurrent AIS in the general population. In this retrospective study, we examined the association of serum β2M and patients with AIS.

## Results

### Summary of the clinical characteristics in patients with acute ischemic stroke, acute spontaneous intracerebral hemorrhage and control subjects

A total of 496 patients were recruited in this study, including 253 healthy control subjects, 202 patients with AIS, and 41 patients with acute spontaneous ICH. Demographics, baseline physical exam characteristics, and laboratory variables are shown in Table 1. All patients were of Chinese Han ethnicity. There were significant differences in age, sex, hypertension, smoking, drinking, systolic blood pressure (SBP), diastolic blood pressure (DBP), homocysteine (HCY), creatinine (Cr), urea, uric acid (UA), total cholesterol (TC), high-density lipoprotein cholesterol (HDL), cystatin C (CysC), C reaction protein (CRP), and β2M among the three groups (*p* < 0.05). However, there were no significant differences in diabetes mellitus, history of stroke, coronary heart disease (CHD), fasting blood glucose (FBG), low-density lipoprotein (LDL), triglyceride (TG), and very low-density lipoprotein cholesterol (VLDL) among the three groups (*p* > 0.05).There were no significant differences in NIHSS,ESRS between AIS and ICH (*p* > 0.05).

**Table 1:** Clinical characteristics between the AIS, ICH and control groups.

Parameters	Control (n = 253)	AIS (n = 202)	ICH (n = 41)	*p* value
Age (year)	65.17 ± 12.81	70.84 ± 11.86^a,b^	59.02 ± 15.48^c^	<0.001
Male sex, n (%)	105 (41.5)	117 (57.9)^a^	29 (70.7)^c^	<0.001
Hypertension, n (%)	118 (46.6)	139 (68.8)^a^	28 (68.3)^c^	<0.001
Diabetes mellitus, n (%)	41 (16.2)	41 (20.3)	8 (19.5)	0.516
History of stroke, n (%)	57 (22.5)	61 (30.2)	10 (24.4)	0.174
CHD, n (%)	29 (11.5)	27 (13.4)	1 (2.4)	0.135
Smoking, n (%)	34 (13.4)	39 (19.3)	13 (31.7)c	0.010
Drinking, n (%)	23 (9.1)	25 (12.4)^b^	10 (24.4)c	0.017
SBP (mmHg)	140.09 ± 19.23	150.87 ± 23.79^a,b^	160.83 ± 25.63^c^	<0.001
DBP (mmHg)	81.09 ± 12.18	85.11 ± 14.91^a,b^	91.90 ± 17.87^c^	<0.001
HCY (μmol/l)	9.90 (4.95)	12.20 (5.53)^a^	12.40 (3.45)^c^	<0.001
FBG (mmol/l)	5.29 (1.43)	5.42 (1.97)	6.09 (1.76)	0.228
CR (μmol/l)	63.89 ± 14.14	75.92 ± 19.05^a,b^	69.90 ± 15.52^c^	<0.001
Urea (mmol/l)	5.11 ± 1.133	5.74 ± 1.73^a,b^	4.91 ± 1.33	<0.001
UA (mmol/l)	289.48 ± 78.02	334.67 ± 84.05^a^	318.51 ± 92.17^c^	<0.001
LDL (mmol/l)	2.50 ± 0.76	2.41 ± 0.73	2.34 ± 0.76	0.230
TG (mmol/l)	1.36 (0.98)	1.47 (1.12)	1.51 ± 0.98	0.310
TC (mmol/l)	4.57 ± 0.92	4.35 ± 0.85^a^	4.39 ± 0.96	0.028
HDL (mmol/l)	1.44 (0.46)	1.40 ± 0.33^a^	1.52 ± 0.40	0.001
VLDL (mmol/l)	0.27 (0.20)	0.30 (0.23)	0.30 ± 0.20	0.237
CysC (mg/l)	0.96 ± 0.19	1.23 ± 0.32^a,b^	0.93 ± 0.22	<0.001
CRP (mg/l)	0.59 (1.77)	1.75 (5.25)^a,b^	0.71 (1.95)	<0.001
β2M (mg/l)	1.50 (0.39)	1.93 (0.67)^a,b^	1.44 ± 0.24	<0.001
NIHSS	—	6 (5)	7 (5)	0.378
ESRS	—	3 (1)	2 (1)	0.407

Quantitative date were expressed as Mean ± SD (normal distribution) or Median (interquartile range) (Non-normal distribution).

a = comparison between the AIS group and the control group, *p* < 0.05; b = comparison between the AIS group and the ICH group, *p* < 0.05; c = comparison between the ICH group and the control group, *p* < 0.05.

CHD, coronary heart disease; SBP, systolic blood pressure; DBP, diastolic blood pressure; HCY, homocysteine; FBG, fasting blood glucose; CR, serum creatinine; UA, uric acid; LDL, low-density lipoprotein cholesterol; TG, triglyceride; TC total cholesterol; HDL, high-density lipoprotein cholesterol; VLDL, very low-density lipoprotein cholesterol; CysC, Cystatin C; CRP, C reaction protein; NIHSS, the National Institute of Health Stroke Scale; ESRS, the Essen Stroke Risk Score.

### Serum β2M level is an independent risk factor for AIS

We used three models to evaluate β2M as the risk factor for AIS (results in Table 1). The results of logistic regression are shown in Table 2. The level of β2M was significantly positively associated with the risk of AIS. In model 1, participants with higher levels of β2M had higher odds of AIS (OR = 9.685, 95% CI = 5.403–17.364). In model 2, based on model 1, we added variables of hypertension, smoking and drinking, and the level of β2M was still significantly associated with the risk of AIS (OR = 9.124, 95% CI = 5.066–16.434). In model 3, based on model 2, we added variables of SBP, DBP, HCY, FBG, CR, Urea, UA, LDL, TC, HDL, CysC and CRP, and the level of β2M was also significantly associated with the risk of AIS (OR = 3.838, 95% CI = 1.715–8.586).

**Table 2:** Risk factors for AIS by binary logistic regression analysis.

	Model 1	Model 2	Model 3
	OR (95% CI)	OR (95% CI)	OR (95% CI)
Age	1.009 (0.990–1.029)	1.010 (0.990–1.029)	1.008 (0.985–1.031)
Sex	0.503 (0.326–0.777)	0.498 (0.313–0.793)	0.610 (0.340–1.095)
Hypertension		2.196 (1.413–3.415)	1.724 (1.062–2.800)
Smoking		0.948 (0.465–1.934)	0.939 (0.438–2.015)
Drinking		1.034 (0.461–2.317)	1.258 (0.531–2.982)
SBP			1.015 (1.002–1.029)
DBP			1.015 (0.994–1.036)
HCY			0.975 (0.944–1.008)
CR			1.001 (0.981–1.022)
Urea			1.047 (0.879–1.246)
UA			1.001 (0.997–1.004)
TC			1.011 (0.751–1.361)
HDL			0.545 (0.252–1.179)
CysC			9.964 (2.359–42.077)
CRP			1.028 (1.002–1.054)
β2M	9.685 (5.403–17.364)	9.124 (5.066–16.434)	3.838 (1.715–8.586)

### Serum β2M was associated with ESRS but not the NIHSS score in patients with AIS

The associations of β2M with the severity of AIS (NIHSS score) and the risk of recurrent cerebral infarction (ESRS) were analyzed using partial correlation analysis. The results showed that β2M levels were significantly and positively correlated with ESRS (*r* = 0.260, *p* < 0.001), but β2M levels were not correlated with the NIHSS score (*r* = 0.083, *p* = 0.239, Table 3).

**Table 3:** Correlation analysis between β2M and NIHSS and ESRS. (n = 202).

Parameters	*r*	*p* value
NIHSS	0.083	0.239
ESRS	0.260	<0.001

### Serum β2M varied in patients with different essen stroke risk scores

**Figure 1:**
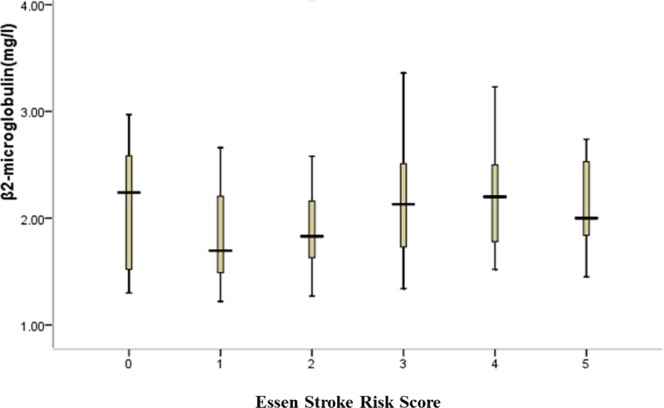
Comparison of β2M in patients with different Essen Stroke Risk Scores. According to the Essen Stroke Risk Score, patients were divided into six groups; the serum β2M levels in the different groups were compared (n = 201).

According to the ESRS, patients with AIS were divided into six groups, and the serum β2M levels in the different groups were compared (the number of patients with a score of 6 points was only one, so we excluded this patient; thus, the total number of subjects was 201) (Fig. 1). The numbers of cases with 0 points, 1 points, 2 points, 3 points, 4 points, and 5 points of ESRS were 7, 24, 62, 66, 33, and 9, respectively, Differences between groups was statistically significant(*p* = 0.007). The level of β2M at 4 points of ESRS was significantly higher than that at 1 point (*p* = 0.001) and 2 points (*p* = 0.006); at the same time, the level of β2M at 3 points of ESRS was significantly higher than that at 1 point (*p* = 0.004) and 2 points (*p* = 0.023) but not significantly different as the level of β2M at 4 points of ESRS compared with 0 points (*p* = 0.401), 5 points (*p* = 0.727). These results may partly indicate that the ESRS increased with the level of β2M. However, the level of β2M at 0 points and 5 points of ESRS did not show this trend, which may be attributed to the sample sizes of patients with 0 points and 5 points being relatively small.

### Serum β2M was associated with TOAST subtypes

Our data showed that the levels of β2M varied with the different subgroups of AIS (TOAST classification) (*p* = 0.018) (Table 4). Kruskal-Wallis analysis was used to compare the levels of β2M in each subtype of TOAST (by extending t-test). Pairwise comparison methods showed that there were significant differences between the large-artery atherosclerosis (LAA) and cardio embolism (CE) groups (*p* = 0.009), the CE and small-vessel occlusion (SAO) groups (*p* = 0.001), and the CE and stroke of other determined etiology (SDE) groups (*p* = 0.016); however, the remaining subtypes were not significantly different (*p* > 0.05) (Table 5).

**Table 4:** Correlation analysis between β2M and TOAST. (n = 202).

Parameters	TOAST					*p* value
	LAA	CE	SAO	SDE	SUE	
β2M (mg/l)	1.96 (0.70)	2.26 (0.75)	1.84 (0.65)	1.80 (0.53)	1.80 (1.10)	0.018

The value of β2M is expressed as median and the interquartile range (IQR). *p* value is a comparison between multiple groups (TOAST subgroups).

**Table 5:** Pairwise comparison between each subtype of TOAST.

TOAST	LAA	CE	SAO	SDE	SUE
LAA	*	0.009	0.307	0.289	0.445
CE	0.009	*	0.001	0.016	0.056
SAO	0. 307	0.001	*	0.534	0.685
SDE	0.289	0.016	0.534	*	0.939
SUE	0.445	0.056	0.685	0.939	*

*p* values are shown.

## Discussion

This cross-sectional study included 202 patients with AIS, 41 patients with ICH, and 253 healthy individuals and investigated the relationships between serum β2M levels and the prevalence of acute cerebral infarction, the severity of infarction (NIHSS score), the subtypes of AIS, and the risk of recurrent stroke (ESRS). At the same time, we compared the serum β2M levels between the AIS group and the intracerebral hemorrhage (ICH) group. Our data revealed that the serum level of β2M was much higher in patients with AIS than in controls and the ICH group, and the results of three binary logistic regression models showed that the level of β2M was significantly associated with the risk of AIS. However, compared to patients with AIS and the control subjects, serum β2M did not increase in the ICH group. Furthermore, the serum β2M level was closely associated with ESRS but not with the NIHSS score, suggesting that the level of β2M was related to the recurrence of AIS. Our data may partly indicate that the ESRS increased with the level of β2M, although we need to expand the sample size in further studies for confirmation. Lastly, the levels of β2M varied with the different subgroups of AIS (TOAST classification), which may provide some clues to the pathogenesis of AIS. These findings indicate that β2M is highly associated with AIS and has some interesting clinical features, which might be a predictor of the risk of AIS/recurrent AIS.

### Causes explaining the high levels of serum β2M in patients with AIS

#### The direct result of AIS

β2M and the MHCI molecule**:** Elevated β2M levels after AIS make us speculate an intriguing phenomenon involving the MHCI molecule’s role during the AIS period. Stroke promotes neurons to express paired immunoglobulin-like receptor B (PirB) and MHCI. MHCI molecules may play a dual role in the inflammatory background, namely, increasing brain tissue damage and limiting neurological function recovery by inhibiting neural plasticity^9^. As the light chain of MHCI, β2M is co-expressed with MHCI molecules^13^ and is also elevated in the model of middle cerebral artery occlusion (MCAO), indicating that the stable expression of MHCI on the cell surface is increased after stroke^9^. In the damaged hemisphere, β2M increases significantly, as it is necessary to stabilize the cell surface to express most MHCI proteins^14^. These results suggest that AIS leads to the increase of β2M, and one reason for the high levels of serum β2M may be the direct result of AIS. However, according to the present research, the mechanism of high-level serum β2M after AIS has seldom been reported. One path may be that β2M can flow into the blood from AIS brain tissues due to the disrupted blood-brain barrier (BBB). Another path may be that the peripheral immune system for MHCI molecules is upregulated after AIS^9^, but further work is needed to verify this hypothesis.

#### Etiological basis of AIS

β2M and inflammation: Recently, β2M has been identified by proteomic profiling as a biomarker of PAD^15^. At same time, it was identified as a risk marker for CHD and stroke in patients with carotid atherosclerosis^5^. The mechanisms may be related to the following factors: As a component of the MHCI complex, β2M is related to inflammation; atherosclerosis is a chronic inflammatory process^16,17^, and inflammation triggers progressive atherosclerosis^17–20^. In particular, the surfaces of lymphocytes and monocytes contain large amounts of β2M, and β2M is synthesized by lymphocytes, regulated by interferon and proinflammatory monocytic cytokines^5^, which may explain the role of β2M in the pathophysiological process of vascular endothelial atherosclerosis. Consistent with the findings of previous studies, our study showed that β2M is associated with AIS, and compared with other subtypes, the levels of β2M were higher in the CE and LAA groups. The possible mechanism is that β2M plays an important role in the formation and development of atherosclerosis (such as carotid atherosclerosis and coronary atherosclerosis (CHD)), which leads to AIS. Meanwhile, our research showed that serum β2M was associated with ESRS. The ESRS score includes many indicators, such as myocardial infarction, cardiovascular diseases, PAD, TIA or cerebral infarction^21,22^. Many indicators (diseases) are the etiological basis of AIS^23–25^ and are associated with chronic inflammation^26–29^. These studies suggest that as markers of (chronic) inflammation, β2M may be the etiological basis of AIS and may be at a high level before the onset of AIS. These studies may be another explanation for the high levels of serum β2M in patients with AIS, and our results further suggested that as a chronic inflammatory biomarker in the body, β2M may have a predictive significance for AIS.

More importantly, our results show that the serum β2M levels in the CE group are significantly higher than those of the other TOAST subtypes, may suggest CE is the strongest reason of elevated β2M level in patients with AIS. With the improvement of diagnostic techniques and classification of AIS^30^, we have a better understanding of CE^31^, literatures report that CE is the most common subtypes of AIS^32,33^, among of them atrial fibrillation (AF) is the most common underlying cause of CE^31,34^. Our results are similar to previous reports: most of our CE patients are with AF, the remaining small number of patients with myocardial infraction or cardiomyopathy, and these diseases are closely associated with chronic inflammation^35–38^. In particular, recent reports have shown that inflammation is directly related to AF which maybe is a direct result of inflammation^39^. It has also been reported that inflammation may be important in the occurrence factor of CE^40^. These views provide a good explanation for our findings. As a biomarker of inflammation, β2M may have more profound significance for the diagnosis and treatment of CE.

### Research significance of β2M in patients with AIS

#### Prevention of AIS

As mentioned above, as a biomarker of inflammation, β2M may be the etiological basis of AIS, reflecting the (chronic) inflammatory status *in vivo*^5,10,41^, which may be of great predictive significance for AIS, especially if the intervention of β2M could reduce the incidence of AIS.

#### Treatment of AIS during the acute stage

Increasing evidence shows that inflammation is one of the main processes leading to the deterioration of the clinical prognosis^42^ of AIS. Meanwhile, inflammation and immunity after stroke are very interesting interactive processes^43^. Under the context of inflammation, neurons increase the expression of PirB and MHCI after stroke and lead to brain damage after ischemia^9^. At the same time, through the autonomic nervous system, the ischemic brain triggers a strong inhibition of the lymphoid organs to avoid further inflammatory injury, but at the expense of an intermittent occurrence of stroke-associated infection^44^. The immune system is closely related to the key events of cerebral ischemic injury and the survival of patients. At present, immunotherapy for AIS has attracted great scientific attention because some signaling pathways have changed after stroke, and blocking some pathways may slow down brain tissue damage, extending the time window of revascularization treatment^45^. As a critical component of MHCI, β2M may provide a new potential target pathway for treating AIS.

### Limitations of this study

First, the low number of subjects is a limitation that may affect our results. Second, multiple medical centers are needed to confirm our findings and improve the accuracy of the estimated glomerular filtration rate to reduce the impact of factors involving renal functions. Finally, we lack the evaluation of the 3-month outcomes of patients with AIS. In the future, we plan to increase the sample size and conduct a multicenter case control study to overcome the limitations of the current study.

## Conclusion

In conclusion, serum β2M is highly associated with acute ischemic stroke, and serum β2M levels are positively correlated with an increased risk of acute ischemic stroke.

## Methods

### Subjects

We collected the clinical data of inpatients with AIS or ICH at the Hospital of Hefei Affiliated Anhui Medical University from September 2015 to October 2016. Patients who were admitted within 3 days after the onset of stroke symptoms were selected. The diagnosis of AIS was confirmed with the diffusion-weighted image sequence of magnetic resonance imaging (MRI), and the diagnosis of acute spontaneous ICH was confirmed with cranial computed tomographic (CT) neuroimaging. Control subjects were collected from outpatients without AIS, transient ischemic attack (TIA), or other neurological diseases during the same period who had normal cranial MRI or CT images.

The exclusion criteria of AIS subjects were stroke history within 6 months, severe brain diseases, serious systemic diseases such as acute/chronic renal dysfunction, endocrine diseases (except diabetes mellitus), cancer, trauma, infectious diseases, and hematological disorders. The exclusion criteria of ICH subjects were patients with acute/chronic renal dysfunction, hematologic disorders, a history of infection within 2 weeks before ICH, a stroke history within 6 months, or a history of malignancy, and patients using immunosuppressant drugs (steroids) or anticoagulation drugs. The exclusion criteria for the healthy controls were the same as above.

### Laboratory and clinical information

The blood samples for laboratory tests were collected on the morning (between 6:00 and 7:00) of the second day after admission with an overnight fast. All samples were sent for testing immediately after collection. Serum β2M was measured with a particle-enhanced turbidimetric immunoassay method. The intra-assay coefficient of variation ranged from 2.4% to 3.8%, and the interassay coefficient of variation ranged from 1.7% to 2.2%. CRP was measured with an immune transmission turbidity method; other biochemical parameters, such as Cr, urea, and TG, were measured with an enzymatic method. All serum biochemical parameters were assayed using an automatic biochemical analyzer (HITACHI Automatic Analyzer 7600-020, Japan).

We collected baseline demographic and clinical information for all participants, including age, sex, and the presence of cerebral vascular risk factors such as hypertension and diabetes. Hypertension was determined by the previous use of an antihypertensive medication, SBP ≥ 140 mmHg or DBP ≥ 90 mmHg. Blood pressure was measured on the admission day using a mercury sphygmomanometer with a supine position of inpatients. Diabetes was determined by the previous use of an antidiabetic medication, fasting blood glucose ≥ 7.0 mmol/l or postprandial blood glucose after 2 h ≥ 11.1 mmol/l.

### Subtypes of ischemic stroke

The categorization of subtypes of ischemic stroke was mainly based on the Trial of ORG 10172 in Acute Stroke Treatment (TOAST)^30^. The TOAST classification denotes five subtypes of ischemic infarction, including LAA, CE, SAO, SDE, and stroke of undetermined etiology (SUE).

### Evaluation of the risk of recurrent stroke

The risk of recurrent stroke for each patient was evaluated according to the Essen Stroke Risk Score (ESRS). The ESRS was derived from cerebrovascular patients in the clopidogrel versus aspirin in patients at the risk of ischemic events (CAPRIE) trial described previously^21,22^. ESRS is a 10-point scale: age 65–75 years (1 point); age >75 years (2 points); arterial hypertension (1 point); diabetes mellitus (1 point); previous myocardial infarction (MI) (1 point); other cardiovascular disease (except MI and atrial fibrillation, 1 point); peripheral arterial disease (1 point); smoker (1 point); and previous TIA or ischemic stroke in addition to qualifying events (1 point). The ESRS score was used to predict the risk of recurrent stroke for each patient^46–48^.

### Evaluation of the severity of AIS

The severity of AIS was assessed according to the National Institute of Health Stroke Scale (NIHSS). NIHSS is widely used to assess the severity of acute ischemic stroke as described previously^49^.

### Statistical analysis

All statistical analyses were conducted with the Statistical Package for the Social Sciences version 19.0 (SPSS, Company, Chicago, IL, USA). Continuous data were tested for normal distributions using the Kolmogorov-Smirnov test. Several continuous variables that followed a normal distribution, such as Cr, UA, TC, LDL, are expressed as the mean ± standard deviation (mean ± SD); other variables that did not follow a normal distribution are presented as the median and the interquartile range (median (IQR)). Categorical variables are expressed as constituent ratios. Differences for continuous variables between groups were assessed by ANOVA or Kruskal-Wallis analysis. Differences in categorical variable distribution between groups were assessed by the χ^2^ test. A pairwise comparison method was used to compare the differences between each group (AIS; ICH and control subjects), LSD was used to analyze quantitative data, and categorical variables were compared using chi-square analysis. Three binary logistic regression analysis models were used to evaluate β2M as the risk factor for AIS. Partial correlation analysis was used to determine the correlations between β2M and NIHSS and ESRS. A *p* value < 0.05 was considered statistically significant.

### Ethical approval and consent to participate

This study was approved by the Research Ethics Committee of the Hospital of Hefei Affiliated Anhui Medical University and had therefore been performed in accordance with the ethical standards laid down in the 1964 Declaration of Helsinki and its later amendments. All patients gave their informed consent prior to their inclusion in the study. The consent was obtained directly from the patient or from a family member or other legal guardian. If a patient was considered incapable of giving informed consent themselves, a family member or other legal guardian was contacted to give informed consent on behalf of the patient. All patients or their key relations were informed of the purpose of the study and signed informed consent. The consent procedures were approved by the ethics committee.

### Consent to publish

All authors read and approved the final manuscript and consent to publish.

## Data Availability

The datasets used and/or analyzed during the current study are available from the corresponding author on reasonable request.

## References

[1] Zumrutdal A (2015). Role of beta2-microglobulin in uremic patients may be greater than originally suspected. World journal of nephrology.

[2] Stanga Z (2013). Factors other than the glomerular filtration rate that determine the serum beta-2-microglobulin level. PloS one.

[3] Filiano AJ, Kipnis J (2015). Breaking bad blood: beta2-microglobulin as a pro-aging factor in blood. Nature medicine.

[4] Kals J (2011). Beta2-microglobulin, a novel biomarker of peripheral arterial disease, independently predicts aortic stiffness in these patients. Scandinavian journal of clinical and laboratory investigation.

[5] Amighi J (2011). Beta 2 microglobulin and the risk for cardiovascular events in patients with asymptomatic carotid atherosclerosis. Stroke.

[6] Joosten, M. M. *et al*. Beta2-microglobulin, cystatin C, and creatinine and risk of symptomatic peripheral artery disease. *Journal of the American Heart Association* **3**, 10.1161/jaha.114.000803 (2014). 10.1161/JAHA.114.000803 PMC4310365 24980133

[7] Smith Lucas K, He Yingbo, Park Jeong-Soo, Bieri Gregor, Snethlage Cedric E, Lin Karin, Gontier Geraldine, Wabl Rafael, Plambeck Kristopher E, Udeochu Joe, Wheatley Elizabeth G, Bouchard Jill, Eggel Alexander, Narasimha Ramya, Grant Jacqueline L, Luo Jian, Wyss-Coray Tony, Villeda Saul A (2015). β2-microglobulin is a systemic pro-aging factor that impairs cognitive function and neurogenesis. Nature Medicine.

[8] Zhu Z (2015). Combination of the Immune Modulator Fingolimod With Alteplase in Acute Ischemic Stroke: A Pilot Trial. Circulation.

[9] Adelson JD (2012). Neuroprotection from stroke in the absence of MHCI or PirB. Neuron.

[10] Prentice RL (2013). Proteomic risk markers for coronary heart disease and stroke: validation and mediation of randomized trial hormone therapy effects on these diseases. Genome medicine.

[11] Prentice RL (2010). Novel proteins associated with risk for coronary heart disease or stroke among postmenopausal women identified by in-depth plasma proteome profiling. Genome medicine.

[12] Rist PM, Jimenez MC, Rexrode KM (2017). Prospective association between beta2-microglobulin levels and ischemic stroke risk among women. Neurology.

[13] Zijlstra M (1990). Beta 2-microglobulin deficient mice lack CD4-8+ cytolytic T cells. Nature.

[14] Huh GS (2000). Functional requirement for class I MHC in CNS development and plasticity. Science (New York, N.Y.).

[15] Wilson AM (2007). Beta2-microglobulin as a biomarker in peripheral arterial disease: proteomic profiling and clinical studies. Circulation.

[16] McDermott MM, Lloyd-Jones DM (2009). The role of biomarkers and genetics in peripheral arterial disease. Journal of the American College of Cardiology.

[17] Schillinger M (2005). Inflammation and Carotid Artery–Risk for Atherosclerosis Study (ICARAS). Circulation.

[18] Schlager O (2007). C-reactive protein predicts future cardiovascular events in patients with carotid stenosis. Stroke; a journal of cerebral circulation.

[19] Sabeti S (2005). Prognostic impact of fibrinogen in carotid atherosclerosis: nonspecific indicator of inflammation or independent predictor of disease progression?. Stroke; a journal of cerebral circulation.

[20] Vidula H (2008). Biomarkers of inflammation and thrombosis as predictors of near-term mortality in patients with peripheral arterial disease: a cohort study. Annals of internal medicine.

[21] A randomised, blinded, trial of clopidogrel versus aspirin in patients at risk of ischaemic events (CAPRIE). CAPRIE Steering Committee. *Lancet (London, England)* **348**, 1329–1339 (1996). 10.1016/s0140-6736(96)09457-3 8918275

[22] Diener HC, Ringleb PA, Savi P (2005). Clopidogrel for the secondary prevention of stroke. Expert opinion on pharmacotherapy.

[23] Shibata T (2015). Prevalence, Clinical Features, and Prognosis of Acute Myocardial Infarction Attributable to Coronary Artery Embolism. Circulation.

[24] Nishino M (1993). Risk factors for carotid atherosclerosis and silent cerebral infarction in patients with coronary heart disease. Angiology.

[25] Tsivgoulis G (2012). Low ankle-brachial index predicts early risk of recurrent stroke in patients with acute cerebral ischemia. Atherosclerosis.

[26] Panhwar, M. S. *et al*. Risk of Myocardial Infarction in Inflammatory Bowel Disease: A Population-based National Study. *Inflammatory bowel diseases*, 10.1093/ibd/izy354 (2018). 10.1093/ibd/izy354 30500938

[27] Golia E (2014). Inflammation and cardiovascular disease: from pathogenesis to therapeutic target. Current atherosclerosis reports.

[28] Elkind MS (2011). Epidemiology and risk factors. Continuum (Minneapolis, Minn.).

[29] Jashari F (2013). Coronary and carotid atherosclerosis: similarities and differences. Atherosclerosis.

[30] Chen PH (2012). Classifying Ischemic Stroke, from TOAST to CISS. CNS neuroscience & therapeutics.

[31] Kernan WN (2014). Guidelines for the prevention of stroke in patients with stroke and transient ischemic attack: a guideline for healthcare professionals from the American Heart Association/American Stroke Association. Stroke.

[32] Hajat C (2011). Incidence of aetiological subtypes of stroke in a multi-ethnic population based study: the South London Stroke Register. Journal of neurology, neurosurgery, and psychiatry.

[33] Gasiorek, P. E. *et al*. Established and potential echocardiographic markers of embolism and their therapeutic implications in patients with ischemic stroke. *Cardiology journal*, 10.5603/CJ.a2018.0046 (2018). 10.5603/CJ.a2018.0046 PMC8084398 29718528

[34] Akiyama H, Hasegawa Y (2018). Awareness of atrial fibrillation in Japan: A large-scale, nationwide Internet survey of 50000 Japanese adults. Geriatrics & gerontology international.

[35] Michniewicz E, Mlodawska E, Lopatowska P, Tomaszuk-Kazberuk A, Malyszko J (2018). Patients with atrial fibrillation and coronary artery disease - Double trouble. Advances in medical sciences.

[36] Teague HL (2017). Unraveling Vascular Inflammation: From Immunology to Imaging. Journal of the American College of Cardiology.

[37] Nishida K, Otsu K (2017). Inflammation and metabolic cardiomyopathy. Cardiovascular research.

[38] Nakamura, M. & Sadoshima, J. Cardiomyopathy in obesity, insulin resistance or diabetes, 10.1113/jp276747 (2019). 10.1113/JP276747 30869158

[39] Van Wagoner DR, Chung MK (2018). Inflammation, Inflammasome Activation, and Atrial Fibrillation. Circulation.

[40] Osawa S (2019). Risk Factors for Hemorrhagic and Cardioembolic Complications of Intracerebral Hemorrhage Associated with Anticoagulants. Journal of stroke and cerebrovascular diseases: the official journal of National Stroke Association.

[41] Shinkai S (2008). Beta2-microglobulin for risk stratification of total mortality in the elderly population: comparison with cystatin C and C-reactive protein. Archives of internal medicine.

[42] Muir KW, Tyrrell P, Sattar N, Warburton E (2007). Inflammation and ischaemic stroke. Current opinion in neurology.

[43] Becker KJ, Buckwalter M (2016). Stroke, Inflammation and the Immune Response: Dawn of a New Era. Neurotherapeutics: the journal of the American Society for Experimental NeuroTherapeutics.

[44] Iadecola C, Anrather J (2011). The immunology of stroke: from mechanisms to translation. Nature medicine.

[45] Fu Y, Liu Q, Anrather J, Shi FD (2015). Immune interventions in stroke. Nature reviews. Neurology.

[46] Maier IL, Bauerle M, Kermer P, Helms HJ, Buettner T (2013). Risk prediction of very early recurrence, death and progression after acute ischaemic stroke. European journal of neurology.

[47] Liu J, Li M, Liu J (2013). Evaluation of the ESRS and SPI-II scales for short-term prognosis of minor stroke and transient ischemic attack. Neurological research.

[48] Thompson DD, Murray GD, Dennis M, Sudlow CL, Whiteley WN (2014). Formal and informal prediction of recurrent stroke and myocardial infarction after stroke: a systematic review and evaluation of clinical prediction models in a new cohort. BMC medicine.

[49] Lyden P (1994). Improved reliability of the NIH Stroke Scale using video training. NINDS TPA Stroke Study Group. Stroke.

